# Progressive supranuclear palsy phenotype as an atypical clinical presentation of Creutzfeldt-Jakob disease: A case report and review of the literature

**DOI:** 10.1016/j.prdoa.2024.100247

**Published:** 2024-03-05

**Authors:** Matteo Costanzo, Flavia Aiello, Anna Poleggi, Pietro Li Voti, Giovanni Fabbrini, Daniele Belvisi

**Affiliations:** a Department of Human Neurosciences, Sapienza University of Rome, Rome, Italy; b Unit of Clinic, Diagnostics and Therapy of the Central Nervous System Diseases, Department of Neuroscience, Istituto Superiore di Sanità, Rome, Italy; c Neurological Centre of Latium, Rome, Italy; d IRCCS Neuromed, Pozzilli (IS), Italy

**Keywords:** Creutzfeldt-Jakob disease, Progressive supranuclear palsy, Atypical presentation

## Abstract

Creutzfeldt-Jakob disease (CJD) is a rare, rapidly progressive neurodegenerative disorder, characterized by the accumulation of abnormal prion proteins in the brain. While CJD has some typical clinical features, its presentation can be quite heterogeneous, particularly in the early stages of the disease, posing challenges in diagnosis. Atypical manifestations of CJD can mimic various neurodegenerative disorders, including atypical parkinsonisms. In this case report, we present an 81-year-old man who exhibited an atypical clinical presentation of sporadic CJD, initially resembling progressive supranuclear palsy (PSP). The patient presented with symmetric parkinsonism, postural instability, and ocular motor dysfunction, accompanied by rapid clinical deterioration. Alongside the case report, we also provide a review of the literature on atypical presentations of CJD as PSP, highlighting the importance of recognizing these manifestations in clinical practice.

## 1 Introduction

Creutzfeldt-Jakob disease (CJD) is a sporadic, transmissible, or familiar neurodegenerative prion disorder characterized by a variable clinical presentation. Here, we report the clinical case of an atypical clinical presentation in an 81-yo-patient affected by sporadic CJD.

## 2 Case presentation

An 81-year-old man with arterial hypertension and a previous prostate cancer presented to the Emergency Department with a 1-month history of gait disturbances with a tendency to fall, often backward, movement slowness, and visual disturbances described as an impairment of visual tracking across a text from one line to another.

**Fig. 1:**
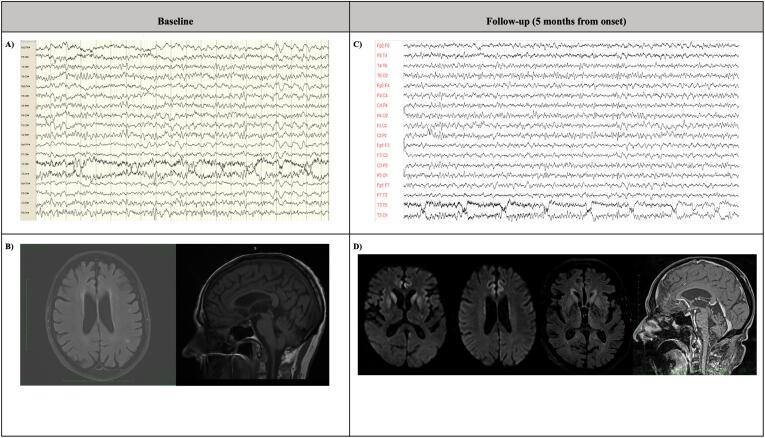
Video-eeg and brain imaging tests at baseline and at follow-up (five-months from the onset). **A)** EEG at baseline: it showed non-specific bilateral centrotemporal theta activity. **B)** Brain MRI (from left to right FLAIR and T1 sequence are displayed) at baseline: it showed chronic vascular encephalopathy; no hummingbird sign in sagittal T1-sequence. **C)** Video-EEG at follow-up: it evidences non-specific diffused theta and delta patterns with a typical repetitive pattern of bilateral synchronous quasi-periodic triphasic waves localized on the posterior regions of the brain. **D)** Brain MRI at follow-up (from left to right DWI, FLAIR and T1 sequences are displayed) demonstrating DWI hyperintensity (bright areas) in the caudate and putamen (hockey-racket sign) and the cortical ribbon. Abnormal hyperintensity is also present on the FLAIR sequence. No hummingbird sign in sagittal T1-sequence. **Abbreviations:**EEG, electroencephalogram; MRI, magnetic resonance imaging.

A complete clinical neurological evaluation revealed bilateral and symmetric parkinsonism with loss of postural reflexes and a limitation of conjugated saccades and pursuit eye movements affecting only the upgaze. The remaining of the neurological examination was unremarkable. The patient scored 28 on the Montreal Cognitive Assessment scale (MoCA), indicating normal cognitive functions. Brain magnetic resonance imaging (MRI) showed chronic vascular encephalopathy **(**Fig. 1**B)**. The electroencephalogram (EEG), performed as a routine investigation in cases of repeated falls of uncertain origin in our clinical setting, showed non-specific bilateral centrotemporal theta activity **(**Fig. 1**A)**. Laboratory tests including a complete blood count, creatinine, blood urea nitrogen, liver function test, serum thyroid-stimulation hormone, lipid panel, and serum folate and vitamin B12 levels were within the normal limits. The physical–chemical, microscopic, and cytological examination of the cerebrospinal fluid (CSF) was also normal. Overall, the main clinical features (symmetric parkinsonism with postural instability and ocular motor dysfunction) suggested the diagnosis of progressive supranuclear palsy (PSP). Treatment up to a daily dose of 300 mg of levodopa was initiated.

After one month there was clinical progression. The neurological examination showed that the patient was disoriented in time and space, dysarthric (**video segment 1**), and was unable to stand or walk due to severe retropulsion. At that time, he begun to use a wheelchair. There was a bilateral symmetric akinetic-rigid syndrome (**video segment 2**) with severe postural instability and a limitation of conjugated saccades and pursuit eye movements affecting only the upgaze (**video segment 5**). In a cognitive screening test using MoCA, the patient reported a score of 16 out of 30, with specific impairment in visual-spatial functions, phonemic verbal fluency, and orientation. Levodopa treatment was ineffective at the administered dose of 300 mg daily. This dosing was carefully chosen considering the patient’s clinical picture, age and potential susceptibility to side effects. Although increasing the dosage might have provided a more comprehensive assessment of the patient’s responsiveness to levodopa treatment, this option was not considered due to the patient’s rapid disease progression, which pointed towards an alternative diagnosis. Positron emission tomography imaging with [(18)F] fluoro-2-deoxy-D-glucose (FDG PET) or dopamine transported single-photon emission computed tomography (DAT-SCAN) were not performed. Because of the rapid clinical worsening and of the atypical clinical presentation, EEG, brain MRI and lumbar puncture were repeated. Brain MRI confirmed the presence of chronic vascular encephalopathy, while the EEG demonstrated slightly slowed background frequencies. A prion RT-QuIC on CSF demonstrated the presence of pathological prion protein, indicating a diagnosis of CJD. In addition, protein 14–3-3 was detected in CSF. Other biomarkers, including tau protein and amyloid, were not assessed in CSF. Genetic testing, performed on blood sample, showed no pathological mutations in the prion protein gene (*PRNP)* while a Val/Val polymorphism was observed at 129 Codon.

Two months later, the patient showed progressive ataxia and myoclonus. At this time, a video-EEG showed the presence of non-specific diffuse theta and delta patterns with a typical repetitive pattern of bilateral synchronous quasi-periodic triphasic waves localized on the posterior regions of the brain (Fig. 1**C**). Brain MRI also suggested finding typical of CJD, demonstrating DWI hyperintensity in the caudate and putamen (hockey-racket sign) and a ribbon-like signal in frontal, temporal, and insular regions (Fig. 1**D**).

The patient ultimately died in hospice seven months after the onset of the disease.

## 3 Discussion

Here, we described a case of a patient affected by CJD with an atypical PSP-like presentation. We thought that at disease onset, the clinical picture was compatible with a diagnosis of possible PSP, according to the Movement Disorder diagnostic criteria [1]. The patient had a slow velocity of vertical saccades (Level of certainty O2 of core clinical features) and a predominantly axial and levodopa-resistant akinetic rigid syndrome (Level of certainty A2 of core clinical features) [1]. Notably, according to the Movement Disorder diagnostic criteria for PSP, a tendency to fall on the pull-test within 3 years from the onset of symptoms is considered a level of certainty P2 of core clinical features [1]. In parallel, laboratory and instrumental exams were unremarkable at disease onset.

However, some red flags were noted at that time. First, the disease course was not typical of PSP because the patient’s condition worsened rapidly over one month, resulting in the loss of independent ambulation and rapid cognitive deterioration. Second, the patient presented with a non-specific vertical gaze palsy, affecting upgaze exclusively, whereas in PSP there is usually a limitation of the range of voluntary gaze in the vertical plane, affecting both up- and downgaze [1].

Supranuclear palsy and parkinsonian signs have been already reported in patients affected by CJD [2], nevertheless a clinical presentation with a phenotype resembling atypical parkinsonism, and in particular PSP, has been rarely described in the initial stage of the disease [3], [4], [5], [6], [7], [8], [9], [10] (Table 1). In a retrospective autopsy study on 180 cases with a clinical diagnosis of PSP evaluated at the Society for PSP brain bank at the Mayo Clinic, Josephs and colleagues described two patients with a pathological diagnosis of CJD [4]. Similarly, to our patient, both cases showed negative findings on MRI and EEG at the initial stage of the disease. Analysis of *PRNP* gene codon 129 polymorphism was not performed in these cases. Shimamura et al. [3] described a case of a patient showing parkinsonism, cerebellar signs, supranuclear palsy, and negative findings on EEG and brain MRI at disease onset [3]. In contrast with our case, levodopa responsiveness was reported, and clinical trajectory was relatively slow, with disease duration longer than 2 years. Molecular genetic analysis revealed homozygosity of methionine/methionine at codon 129 of the prion protein. The presence of homozygosity for methionine at codon 129, without pathological mutation of the prion protein gene, was also reported by other groups [8], [9], [11]. Petrovic et al. [11] described one patients who had vertical supranuclear palsy, bradykinesia, axial rigidity, cognitive impairment and Pisa syndrome. As in our case, MRI and EEG were not typical for CJD at the onset of the disease. Hamaguchi et al [8], described two cases who had parkinsonian signs associated to non-motor symptoms (sleep, psychiatric and autonomic disorders). In one case, pyramidal symptoms were also present.

**Table 1:** Characterisation of the cases of sCJD resembling PSP described in literature.

	Age	Disease’s duration (months)	Disease’s features				Instrumental findings		CSF	Levodopa’s responsiveness	Polymorphisms
			Myoclonus	Cognitive impairment	Parkinsonism	Others	EEG	MRI			
**Shimamura et al., 2009**	56	> 24	–	+	Postural instability, vertical gaze palsy, bradykinesia	Dysarthria	Aspecific (Diffused theta waves)	Compatible (2 months later)	- (14–3-3 protein)	Responsive	No polymorphism
**Josephs et al., 2004**	70	29	–	+ (After 2 y)	Postural instability, hypophonia, axial rigidity, resting tremor	Visual difficulties, apraxia	Aspecific	Incompatible	Not performed	Unresponsive	Not specified
	75	10	–	+ (After 7 months)	Postural instability	Visual difficulties	Aspecific	Incompatible	Not performed	Unresponsive	Not specified
**Lourenço Rosa et al., 2022**	66	4	–	+ (1 months)	Postural instability, apraxia, upward gaze limitation, bradykinetic-rigid syndrome, hypomimia,	Explosive speech, ataxia, dysarthria	Aspecific (Diffused slowing)	Compatible	+ (14–3-3 protein)	Not specified	Not specified
**Matej et al, 2012**	62	16	+ (Later than onset)	+	Alteration of gait, hypophonia, slow saccades and upward gaze limitation, rigidity	Mood disorders, dysarthria, dysmetria, sensory-motor neuropathy, choreatic movements of the arms	Specific (Later: triphasic complexes)	Compatible (Later than onset)	+ (14–3-3 protein)	Unresponsive	R208H + Val/Val at codon 129
**Petrovic et al., 2012**	71	18	Not specified	+	Postural instability, bradykinesia, vertical gaze limitation,	Bilateral sensorineural hearing loss,, brisk jaw jerk, dysarthria, intermittent jerky postural tremor	Not specified	Incompatible	Not specified	Unresponsive	Met/Met at codon 129
**Prasad et al., 2007**	74	Not specified	+ (At least after 3 months)	+ (After 3,5 months,, as impaired memory and visual allucinations)	Gait instability supranuclear upgaze palsy, slow saccades in downgaze, hypometric saccades	Fasciculations reduction of all type of sensation, slowness, dysmetria, ataxia	Specific	Compatible	± (14–3-3 protein at 3 months)	Not specified	Not specified
	61	15	Not specified	+ (Progressive deterioration in the last phases)	Limited upgaze, slowed vertical saccades (>downgaze), mild facial masking, hypophonia, slurred speech.	Ataxia, binocular horizontal diplopia, dysarthria, rebound nystagmus and square wave jerks, mild exophoria, brisk symmetrical reflexes	Aspecific	Incompatible	Ambiguous result for 14–3-3 protein		
**Rowe et al., 2007**	62	4	–	+ (In the last phases)	No vertical saccades, fragmentation of pursuit eye movements, increased axial tone, postural instability.	Dysarthria, dysphagia, language difficulties	Aspecific	Incompatible	- (14–3-3 protein)	Unresponsive (200 mg x 3/die)	A133V, MM, carrier E219K
**Huber et al., 2007**	67	19	± (Later than onset)	± (after 1 month)	Postural instability, vertical gaze palsy	Dysphagia, Ideomotor apraxia	Aspecific	Compatible (Later than onset)	+ (14–3-3 protein)	Unresponsive	Not specified
**Hamagushi et al., 2005**	49	30	±	±	Extrapyramidal signs	Insomnia, psychiatric symptoms, pyramidal signs, autonomic symptoms, akinetic mutism	Aspecific (slowing)	NA	NA	Not specified	MM2
	64	53	±	±	Extrapyramidal signs	Visual symptoms, autonomic symptoms, psychiatric symptoms, akinetic mutism	Aspecific (slowing)	NA	NA	Not specified	MM2

**Abbreviations:** EEG, electroencephalogram; MRI, magnetic resonance imaging; NA, not available; CSF, cerebrospinal fluid.

We therefore report the first case of a patient with sporadic CJD with valine homozygosity at codon 129 of the prion protein manifesting a clinical phenotype resembling PSP at the initial stage of the disease. A similar presentation at disease onset was previously reported only in 1 patient affected by a genetic form of CJD with R208H mutation in the *PRNP* gene and a valine homozygosity at codon 129 [12]. MRI and EEG in the patient described by Matěj and colleagues were normal at the initial stages of the disease, whereas differently from our patient disease duration was relatively longer with about one year of time interval between the disease onset and the development of myoclonus.

The pathophysiological mechanisms underlying the phenotypic overlap between CJD and PSP are complex and not fully understood. In the study of Josephs et al., the neuropathological examination performed on two CJD patients presenting with PSP-like features revealed significant neuronal loss and gliosis with spongiform changes in the substantia nigra, suggesting a dopaminergic nigrostriatal pathway dysfunction [4]. However, case reports investigating the dopamine transporter system in sporadic CJD, by using DAT-SCAN, have yielded conflicting results. Presynaptic dopaminergic depletion has been reported in isolated cases of CJD patients [13], [14]. On the other hand, Kim et al [15] described a patient with sporadic CJD exhibiting rapid bilateral parkinsonism yet having preserved dopaminergic function [15].

Overall, a hypothesis that might explain the phenotypic heterogeneity observed in CJD and also applicable in other neurodegenerative disorders is based on network-based neurodegeneration, where differential spatial and temporal propagation of disease-specific proteins determine variable clinical manifestations [16]. In this context, the pattern and distribution of prion protein deposition may be specific to the subtype of CJD. For instance, VV2 subtype has been associated with plaque-like and perineuronal immunoreactivity, especially prominent in the deep cortical layers, basal ganglia, and cerebellum [16], [17]. However, while it’s tempting to associate the above-mentioned discrepancies with differences in *PRNP* gene polymorphism, which have been known to influence prion-strain properties, including locations, structure and seeding capacities [18], [19], it is essential to note that current evidence does not clearly establish discernible differences in dopaminergic function across PRNP genotypes. Further research would be required to validate or refute this potential correlation. Moreover, future neuropathological studies in atypical presentations of CJD are needed to clarify the relationship between molecular subtypes of the disease and clinical phenotypes at the initial stage.

In conclusion, this case report underlines the importance of considering CJD in patients manifesting signs of atypical parkinsonism, especially when the clinical course of the disease is rapidly progressive. This consideration is crucial even when there are no initial laboratory or instrumental findings indicative of CJD, as these might only become apparent later in the disease’s timeline. Therefore, repeating diagnostic tests throughout the disease’s clinical history is advisable to ensure a prompt and accurate diagnosis.

## CRediT authorship contribution statement

**Matteo Costanzo:** Conceptualization, Data curation, Writing – original draft, Investigation. **Flavia Aiello:** Data curation, Writing – original draft, Investigation. **Anna Poleggi:** Data curation, Writing – original draft, Investigation. **Pietro Li Voti:** Conceptualization, Data curation, Investigation. **Giovanni Fabbrini:** Writing – review & editing, Supervision. **Daniele Belvisi:** Conceptualization, Writing – review & editing, Supervision.

## Declaration of competing interest

The authors declare that they have no known competing financial interests or personal relationships that could have appeared to influence the work reported in this paper.
